# Obesity in British children with and without intellectual disability: cohort study

**DOI:** 10.1186/s12889-016-3309-1

**Published:** 2016-07-27

**Authors:** Eric Emerson, Janet Robertson, Susannah Baines, Chris Hatton

**Affiliations:** 1 Centre for Disability Research, Lancaster University, Lancaster, UK; 2 Centre for Disability Research and Policy, University of Sydney, Sydney, New South Wales Australia

## Abstract

**Background:**

Reducing the prevalence of and inequities in the distribution of child obesity will require developing interventions that are sensitive to the situation of ‘high risk’ groups of children. Children with intellectual disability appear to be one such group. We aimed to estimate the prevalence of obesity in children with and without intellectual disability in a longitudinal representative sample of British children and identify risk factors associated with obesity at age 11.

**Methods:**

Information was collected on a nationally representative sample of over 18,000 at ages 9 months, 3, 5, 7 and 11 years. We used UK 1990 gender-specific growth reference charts and the LMS Growth programme to identify age and gender-specific overweight and obesity BMI thresholds for each child at ages 5, 7 and 11 years.

**Results:**

Children with intellectual disabilities were significantly more likely than other children to be obese at ages five (OR = 1.32[1.03–1.68]), seven (OR = 1.39[1.05–1.83]) and eleven (OR = 1.68[1.39–2.03]). At ages five and seven increased risk of obesity among children with intellectual disabilities was only apparent among boys. Among children with intellectual disability risk of obesity at age eleven was associated with persistent maternal obesity, maternal education, child ethnicity and being bullied at age five.

**Conclusions:**

Children with intellectual disability are a high-risk group for the development of obesity, accounting for 5–6 % of all obese children. Interventions to reduce the prevalence and inequities in the distribution of child obesity will need to take account of the specific situation of this group of children.

## Background

Reducing child obesity is a key public health priority internationally [1–3] and in many high income countries [4–7]. Reducing the prevalence and inequities in the distribution of child obesity will require developing interventions that are sensitive to the situation of ‘high risk’ groups of children [8]. Children with intellectual disability appear to be one such high risk group. Intellectual disability refers to a significant general impairment in intellectual functioning that is acquired during childhood [9]. Estimates of the prevalence of intellectual disability derived from epidemiological studies vary widely, with pooled estimates for children suggesting a point prevalence of 1.83 % (95 % CI confidence intervals 1.52–2.14 %) [10]. However, it has been estimated by Public Health England that approximately 3 % of the child population of England have an intellectual disability [11].

The available evidence suggests that children with disabilities generally [12–14], and children with intellectual disability specifically, are at increased risk of overweight and/or obesity [15]. The increased risk of obesity among children with intellectual disability has been reported in a diverse range of countries including Australia [16, 17], France [18, 19], Japan [20], Korea [21], Taiwan [22], the UK [23–25] and the USA [26–28]. The only exception to this pattern being one report of lower rates of obesity among children with intellectual disabilities in Hong Kong [29].

Among children with intellectual disabilities higher rates of obesity have been reported among: older children [16–18, 20–22, 25, 26]; girls [18, 20, 21, 30]; boys [26]; children with Down syndrome [18]; children taking psychoactive medication [18]; children living in poorer families [16]; children living in more socially deprived neighbourhoods [16]; and children in high income countries [30, 31]. However, only the association between obesity and age has been reported with any degree of consistency [15]. Among adults with intellectual disabilities higher rates of obesity have been reported among women, people with Down’s syndrome, people with less severe intellectual disabilities, people living in less restrictive residential environments and people living in more socially deprived neighbourhoods [32–34].

The existing literature does, however, have two significant limitations. First, the majority of studies have relied on convenience samples (e.g., children attending special schools [18, 20, 21, 24, 25, 28, 29], children participating in Special Olympics [26, 30, 31, 35]) that are unlikely to be representative of the wider population of children with intellectual disabilities. Second, the vast majority of studies have employed cross-sectional designs. The aims of the present paper are to redress these deficiencies in the existing evidence-base by: (1) estimating the prevalence of overweight and obesity across early and middle childhood in children with and without intellectual disability in a longitudinal representative sample of British children; (2) identify risk factors associated with overweight and obesity in children with intellectual disability at age 11.

## Method

The UK’s Millennium Cohort Study (MCS) is the fourth in the series of British birth cohort studies. It aims to follow throughout their lives a cohort of over 18,000 children born in the UK between 2000 and 2002. MCS data are managed by the Centre for Longitudinal Studies at the University of London (www.cls.ioe.ac.uk/) and are available to researchers registered with the UK Data Service (www.ukdataservice.ac.uk) (www.esds.ac.uk). Full details of the design of MCS are available in a series of reports and technical papers [36–42] key aspects of which are summarised below.

### Sampling

Participant families were randomly selected from Child Benefit Records, a non means-tested welfare benefit available to all UK children at the time the cohort was established. Sampling was geographically clustered to include all four countries of the UK (England, Wales, Scotland, Northern Ireland), and disproportionately stratified to over-sample children from ethnic minority groups and disadvantaged communities [43]. Children and families were drawn from 398 randomly selected electoral wards in the UK. The first survey (MCS1) took place when children were nine months old and included a total of 18,552 families. Children were followed up at ages three (MCS2; 15,590 families, 78 % response rate), five (MCS3; 15,246 families, 79 % response rate), seven (MCS4; 13,857 families, response rate) and eleven (MCS5; 13,287 families, 69 % response rate). For each family, information was collected on the target child falling within the designated birth date window. For multiple births (e.g., twins, triplets) information was collected on all children. To avoid the statistical problems associated with the clustering of multiple births within households, the present analyses are restricted to the first named target child in multiple birth households. All analyses used sampling weights provided with MCS data to adjust for the initial sampling design and biases in recruitment and retention at specific ages.

### Identification of children with intellectual disability

Child cognitive ability was assessed at age three using the Bracken School Readiness Assessment [44] and Naming Subscale of the British Ability Scales (BAS) [45], selected subscales of the BAS at ages five and seven, and the NFER Progress in Maths test at age seven [46]. At age 11 children were given three cognitive tests; verbal similarities (BAS), the Spatial Working Memory task and the Cambridge Gambling task, both from the Cambridge Neuropsychological Test Automated Battery. Of the age eleven tests, only verbal similarities is closely related to traditional measures of IQ.

For ages five and seven we extracted the first component (‘g’) from a principal component analysis of all age-standardised subscale/test scores [47]. The first component accounted for 63 % of score variance at age seven and 55 % of score variance at age five. We identified children as having intellectual disability if they scored two or more standard deviations below the mean on the first principal component at age seven (*n* = 419 [3.3 %] of 12,820 children for whom test results were available).

If cognitive test scores were missing at age seven, we identified children as having intellectual disability if they scored two or more standard deviations below the mean on the first principal component at age five (*n* = 146 [6.5 %] of 2,250 children). If cognitive test scores were missing at age five and at age seven, we identified children as having intellectual disability if they scored two or more standard deviations below the mean on the Bracken School Readiness Assessment at age three (*n* = 49 [4.4 %] of 1105 children). If Bracken scores were not available, we identified children as having intellectual disability if they scored two or more standard deviations below the mean on the BAS Naming Subscale at age three (*n* = 54 [7.6 %] of 711 children). This process allowed us to classify intellectual disability on the basis of cognitive test scores for 99.1 % of children participating at age seven.

For 125 children no cognitive test results were available at any age. Interviewers did not administer the assessments if the child ‘has a learning disability/serious behavioural problem (e.g., severe ADHD, autism) which prevents them from carrying out the assessments’, ‘is unable to respond in the required manner for each assessment, e.g., reading, writing, manipulating objects’, ‘is not able to speak or understand English (or Welsh if applicable)’ or if consent and co-operation were not forthcoming. For these children we identified intellectual disability on the basis of parental report at age seven. A child was identified as having learning disabilities if both of the following two criteria were met: (1) the child was reported to be receiving special education due to their ‘learning difficulty’ (the term used in educational services in the UK to refer to intellectual disability); (2) the child was reported to have ‘great difficulty’ in all three areas of reading, writing and maths. This led to the identification of another 11 children as having intellectual disability (8.8 % of children for who no test scores were available).

Finally, we used the normalised verbal similarities standard score at age eleven to attempt to address potential errors in classification in the W2-4 variables. Specifically, all children who had been identified as having intellectual disability who scored at or above the population mean on verbal similarities at age eleven were reclassified as not having intellectual disability. Similarly, all children identified as not having intellectual disability but who scored three or more standard deviations below the population mean on verbal similarities at age eleven were reclassified as having intellectual disability.

This procedure led to the identification of 647 of the 18,495 (3.5 %) children participating at Wave 1 where the child’s mother was the primary informant as having intellectual disability. As expected, boys were significantly more likely than girls to be identified as having intellectual disability (4.3 % vs 2.6 %; OR = 1.67, 95 % CI 1.42–1.96).

### Overweight & obesity

Measurements of child height and weight were taken and BMI calculated at ages three (MCS2), five (MCS3), seven (MCS4) and eleven (MCS5). MCS data releases include categorical measures of overweight and obesity based on the International Task Force childhood obesity reference charts and cut-off points [48]. At ages three and five, 5 % of cohort members were identified as obese [49, 50]. However, studies have suggested that: (1) the international reference lacks specificity due to the sample size used to define the population; (2) their use may lead to the under-reporting of rates of obesity; and (3) international reference charts may ignore population differences in the relationship between BMI and adiposity [51]. As a result, more recent studies of child obesity using MCS data have used UK-specific growth reference charts to recalculate overweight and obesity from BMI, age and gender data [52].

In the present study we used UK 1990 gender-specific growth reference charts and the LMS Growth programme to identify age and gender-specific overweight and obesity BMI thresholds for each child at ages 5, 7 and 11 years [53, 54]. The use of UK 1990 BMI growth reference charts to calculate obesity is not recommended under 4 years of age [55]. Children whose BMI fell at or above the 85th percentile of the UK 1990 reference population were defined as overweight, and those at or above the 95th percentile were defined as obese [55, 56].

### Predictor variables

Previous research undertaken with the MCS has reported that increased risk of overweight and obesity was associated with a range of variables including ethnicity, higher birth weight, early introduction of solid foods, missing breakfast, sedentary lifestyle, maternal smoking (including during pregnancy), parental overweight (including pre-pregnancy overweight), lone motherhood, maternal employment, low parental educational attainment, low income, material hardship, living in more socially deprived neighbourhoods [23, 49, 50, 52]. The following potential predictor variables were extracted from MCS data collected in Waves 1–4.

#### Child ethnicity

Child ethnicity was based on parental report and coded using the six category UK Census scheme: White; Mixed Ethnicity; Indian; Pakistani or Bangladeshi; Black or Black British; Other.

#### Child birth weight

Parental report of child birth weight was collected at Wave 1. These data were recoded into a binary measure of higher birth weight based on falling within the upper quartile of the weighted sample distribution (> 3.70 kg).

#### Child nutrition

At Wave 1 (9 months) information was collected from the main parental informant on the age at which the child first had solid foods. These data were recoded into a binary measure of introduction of solid foods in the first three months of life [52].

At Waves 3 and 4 information was collected from the main parental informant on: (1) the number or portions of fruit eaten daily; (2) the main type of between-meal snacks the child eats; and (3) the number of days the child has breakfast. Following inspection of the distribution of these data, binary measures were derived of: (1) low levels of fruit consumption (less than two portions per day); (2) higher calorie snacks (the main type of snack being crisps, cakes or biscuits, sweets); (3) child skips breakfast one or more days per week.

#### Child participation in sport & physical activity

Parental report of child participation in sport was collected at Waves 3 and 4. Following inspection of the distribution of these data, binary measures of low levels of participation were derived based on participation on less than one day per week (age 5, Wave 3) and participation on less than two days per week (age 7, Wave 4). Parental report of child participation in physical activity sport was collected at Wave 4. Following inspection of the distribution of these data, a binary measure of low participation was derived based on participation on less than five days per week.

#### Child tv watching and playing computer games

Parental report of the time the child spends watching TV was collected at Waves 2–4. Parental report of the time the child spends playing computer games was collected at Waves 3 and 4. Binary measures of high levels of TV watching or computer game playing were derived based on watching/playing for three or more hours daily. At Wave 4 information was also collected on whether the child had a TV in their bedroom.

#### Child exposure to bullying

The Strengths and Difficulties Questionnaire [57] was completed by parents at Waves 2–4. One item ‘*Picked on or bullied by other children*’ was used to identify exposure to bullying at ages three, five and seven.

#### Maternal obesity

Maternal BMI was calculated from self-reported height and weight at each of the four MCS waves and retrospectively at Wave 1 for pre-pregnancy weight. Obesity was defined as a BMI of 30 or more. Maternal obesity status over time was coded as never, once or twice, three or more times.

#### Maternal smoking

Information was collected at each Wave on whether the child’s mother was a current smoker. Following inspection of the distribution of these data a binary measure of maternal smoking was derived based on being a current smoker at two or more of the four waves of data collection.

#### Maternal educational attainment

Highest level of educational attainment across waves was coded according to National Vocational Qualifications (NVQ) categories.

- No qualifications or NVQ Level 1: Competence that involves the application of knowledge in the performance of a range of varied work activities, most of which are routine and predictable (equivalent to one General Certificate of Secondary Education (GCSE) at grade D-G).
- NVQ Level 2: Competence that involves the application of knowledge in a significant range of varied work activities, performed in a variety of contexts. Collaboration with others, perhaps through membership of a work group or team, is often a requirement (equivalent to one GCSE at grade A*-C).
- NVQ Levels 3–5: NVQ Level 3 requires competence that involves the application of knowledge in a broad range of varied work activities performed in a wide variety of contexts, most of which are complex and non-routine. There is considerable responsibility and autonomy and control or guidance of others is often required (equivalent to 1–5 Advance Level certificates at grades A*-C).
- Overseas qualifications only.

#### Maternal employment

Information on maternal employment and hours worked was collected at each Wave. High levels of hours worked were defined at each wave as working for more than 20 h per week [52].

#### Single parent family

Information on household composition was collected at each Wave. Following inspection of the distribution of these data a single binary measure of single parent family was derived based on being a single parent family at any of the four waves of data collection.

#### Household income poverty

Information on household income was collected at each Wave. These were adjusted for household composition using the modified OECD equivalisation scale [58] and used to define household income poverty (equivalised household income falling 60 % below the population median) [59]. Following inspection of the distribution of these data a single binary measure of household income poverty was derived based on a household being in income poverty at any of the four waves of data collection.

#### Area deprivation

At each wave postcode data were linked to country-specific area-based measures of multiple deprivation [60]. These were recoded into a binary measure of living in an area characterised by high levels of local area deprivation based on living in an area in the lowest quintile of neighbourhoods in a given country at all four waves of data collection (vs. not).

### Approach to analysis

In the first stage of analysis we used simple bivariate descriptive statistics to estimate the prevalence of overweight and obesity at ages five, seven and eleven for children with and without intellectual disability. In the second stage of analysis we used simple bivariate descriptive statistics to estimate the persistence of obesity between successive waves of data collection for children with and without intellectual disability.

In the third stage of analysis we examined bivariate associations between the predictor variables listed above and obesity at age 11 in the full sample. Missing data on predictor variables was imputed using multiple imputation routines in SPSS 20 to create five parallel data sets. The results of pooled analyses were used to exclude predictor variables from subsequent analyses if they had no statistically significant association with child obesity. This led to the exclusion of all measures of maternal employment and at Wave 4 indicators of having a TV in the child’s bedroom, the number of days of physical activity and the main type of between-meal snack. In the fourth stage of analysis we determined the strength and statistical significance of the bivariate association between remaining predictor variables and child obesity at age 11 for children with and without intellectual disability.

In the fifth stage of analysis we used multivariate logistic regression to determine the unique strength and statistical significance of the association between predictor variables that were either significantly associated or showed moderate or greater effect sizes in relation to obesity at age 11 among children with intellectual disability. In the final stage of the analysis we applied the model from the fifth stage to the sub-sample of children without intellectual disability. The aim of this stage of the analysis was to investigate whether there were any marked difference in the strength of association between predictor variables and obesity that were significant for children with intellectual disability when applied to children without intellectual disability. We did not undertake any further analyses of the non-intellectual disability subsample as data has been reported elsewhere on the full sample, the results of which are driven by the non-intellectual disability subsample [49, 50, 52]. In the final two stages of the analyses missing data on predictor variables was imputed using multiple imputation routines in SPSS 20 to create five parallel data sets. The results of pooled analyses were reported.

All analyses used appropriate wave-specific weights to take account of biases in the sampling frame (e.g., oversampling of households in Scotland, Wales and Northern Ireland), initial recruitment and attrition over time.

## Results

### The prevalence of child obesity

Mean BMI, height and weight by age, intellectual disability status and gender are presented in Table 1. Table 2 presents estimated prevalence rates for overweight and obesity at ages 5, 7 and 11 years for boys and girls with and without intellectual disability. Boys with intellectual disability were at significantly increased risk of obesity, when compared to boys without intellectual disability, at all ages. Girls with intellectual disability were at significantly increased risk of obesity, when compared to girls without intellectual disability at age 11. They were also at significantly increased risk of overweight at age 7. Overall, children with intellectual disability were not at significantly greater risk of overweight at any age. They were, however, at significantly greater risk of obesity at ages 7 and 11. At age 11, 5 % of all obese children were children with intellectual disability.

**Table 1:** BMI, height and weight (mean, standard deviation and sample size) by age, intellectual disability status and gender

Age	ID		No ID	
	Boys	Girls	Boys	Girls
5 years	*N* = 325	*N* = 203	*N* = 7274	*N* = 7112
Height (cm)	109.6 (5.6)	107.8 (5.8)	111.2 (5.0)	110.3 (4.9)
Weight (kg)	20.1 (4.1)	19.1 (3.0)	20.4 (3.3)	19.9 (3.1)
BMI	15.3 (5.2)	15.6 (4.2)	16.1 (2.8)	16.1 (2.6)
7 years	*N* = 282	*N* = 174	*N* = 6380	*N* = 6217
Height (cm)	121.4 (5.5)	120.6 (6.7)	124.2 (5.6)	123.3 (5.5)
Weight (kg)	25.0 (5.3)	25.2 (6.2)	25.7 (4.9)	25.4 (4.9)
BMI	16.9 (2.8)	17.1 (3.1)	16.6 (2.3)	16.6 (2.4)
11 years	*N* = 319	*N* = 204	*N* = 7476	*N* = 7009
Height (cm)	144.1 (8.3)	143.7 (8.5)	146.0 (7.0)	146.8 (7.5)
Weight (kg)	41.2 (11.7)	42.6 (11.0)	40.8 (9.8)	42.1 (10.2)
BMI	19.7 (4.4)	19.4 (4.4)	19.0 (3.5)	19.4 (3.7)

**Table 2:** Estimated prevalence of overweight and obesity at ages 5, 7 and 11 for boys and girls with and without intellectual disability

	Overweight				Obese				Overweight or Obese			
	ID	Not ID	OR (with 95 % CI)/p	% with ID	ID	Not ID	OR (with 95 % CI)/p	% with ID	ID	Not ID	OR (with 95 % CI)/p	% with ID
*Age 5*												
Boys	13.5 %	15.7 %	0.82 (0.59–1.13)	3.6 %	17.2 %	12.8 %	1.41* (1.05–1.89)	5.7 %	30.2 %	28.6 %	1.08 (0.85–1.38)	4.5 %
Girls	16.8 %	13.0 %	1.35 (0.93–1.97)	3.5 %	11.8 %	11.0 %	1.08 (0.70–1.67)	3.0 %	28.6 %	24.0 %	1.27 (0.93–1.72)	3.3 %
Total	14.6 %	14.6 %	1.02 (0.80–1.30)	3.6 %	15.1 %	11.9 %	1.32* (1.03–1.68)	4.5 %	29.5 %	26.3 %	1.17 (0.97–1.42)	4.0 %
*Age 7*												
Boys	10.2 %	12.2 %	0.82 (0.52–1.28)	2.9 %	21.4 %	14.3 %	1.64** (1.17–2.28)	5.0 %	31.6 %	26.5 %	1.29 (0.96–1.72)	4.1 %
Girls	20.8 %	11.4 %	2.04** (0.35–3.07)	4.2 %	11.8 %	12.3 %	0.95 (0.57–1.59)	2.3 %	32.6 %	23.8 %	1.55* (1.09–2.21)	3.2 %
Total	14.5 %	11.8 %	1.26 (0.94–1.70)	3.5 %	17.5 %	13.3 %	1.39* (1.05–1.83)	3.8 %	32.0 %	25.1 %	1.40** (1.12–1.76)	3.7 %
*Age 11*												
Boys	10.7 %	15.2 %	0.67* (0.47–0.96)	2.9 %	31.0 %	22.0 %	1.59*** (1.25–2.03)	5.7 %	41.8 %	37.2 %	1.21 (0.97–1.52)	4.6 %
Girls	17.2 %	13.9 %	1.28 (0.89–1.85)	3.4 %	31.2 %	20.2 %	1.79*** (1.33–2.42)	4.3 %	48.3 %	34.1 %	1.80*** (1.36–2.38)	3.9 %
Total	13.2 %	14.6 %	0.89 (0.69–1.16)	3.2 %	31.1 %	21.2 %	1.68*** (1.39–2.03)	5.0 %	44.4 %	35.7 %	1.44*** (1.20–1.71)	4.3 %

ORs in bold indicate medium or large effect size

*ID* intellectual disability, *OR* odds ratio, *p* probability, *CI* confidence interval

* *p* < 0.05, ** *p* < 0.01, *** *p* < 0.001

### The persistence of child obesity

Table 3 presents estimated persistence rates for obesity between ages 5, 7 and 11 years for children with and without intellectual disability. Column three presents estimates of the prevalence of persistent obesity (obese at time 1 and at time 2) within each of the two samples. Between ages five and seven and seven and eleven there were no statistically significant differences in the estimated prevalence of persistent obesity between children with and without intellectual disability. Between the ages of five and eleven, persistent obesity was marginally higher among children with intellectual disability (z = 2.32, *p* < 0.05).

**Table 3:** The persistence of obesity in British children with and without intellectual disability

Time period	Group	Overall prevalence (with 95% CI) of persistent obesity (obese at time 1 and time 2)	Relative risk (with 95 % CI and p) of obesity at time 2 if obese at time 1
Age 5 to 7	ID	7.6 % (4.8–10.4 %)	5.69*** (3.99–8.91)
	Not ID	8.3 % (7.8–8.8 %)	13.01*** (11.92–14.20)
Age 5 to 11	ID	11.2 % (8.3–14.1 %)	3.11*** (2.45–3.95)
	Not ID	8.2 % (7.7–8.6 %)	4.94*** (4.68–5.21)
Age 7 to 11	ID	11.6 % (8.1–15.1 %)	3.06*** (2.34–4.00)
	Not ID	10.6 % (10.0–11.1 %)	7.28*** (6.88–7.71)

Relative risks in bold indicate medium or large effect size

*ID* intellectual disability, *p* probability, *CI* confidence interval

*** *p* < 0.001

Column four presents estimates of the relative risk of a child being obese at time 2 (e.g., 7 years old) if they were obese at time 1 (e.g., 5 years old). The comparison in the calculation of relative risk in these instances is the probability of a child from the same group being obese at time 2 if they were not obese at time 1. In all instances and for each group, obesity at a later age was significantly and markedly greater (3–11 times more likely) among children who were already obese. At all ages, there was a significantly stronger association between obesity at times 1 and 2 for children without intellectual disability.

### Predictors of child obesity

As can be seen in Table 2, among children with intellectual disability rates of obesity were higher among boys than girls at age five (OR = 1.55 [0.93–2.58]) and seven (OR = 2.03 [1.11–3.72]). Similar, but weaker, associations were also seen among children without intellectual disability (age five OR = 1.19 [1.08–1.32], age seven OR = 1.18 [1.06–1.31]). By age eleven, however, the association between male gender and obesity was no longer apparent among children with intellectual disability (OR = 0.99 [0.68–1.45]), but did persist among children without intellectual disability (OR = 1.11 [1.03–1.21]).

Table 4 presents information on the strength and statistical significance of the bivariate association between predictor variables and child obesity at age 11 for children with and without intellectual disability. As described above, predictors were only included if there was a statistically significant association between the predictor and obesity at age 11 in the full (combined) sample. Given that children without intellectual disability made up 96.5 % of the full sample, it is unsurprising that there were statistically significant associations between all predictors and obesity at age 11 among children without intellectual disability. Statistically significant associations between predictor variables and child obesity at age 11 were much rarer among children with intellectual disability, only being evident for maternal obesity, maternal educational attainment (overseas qualifications), child ethnicity (Black/Black British) and (somewhat paradoxically) high levels of fruit consumption at age five. The latter result should be treated with some caution as: (1) the association was no longer statistically significant in the subsequent multivariate analyses (Table 4); (2) the effect size is small;[61] and (3) opposite (though non-significant) associations between fruit consumption and obesity were evident at age seven. While not statistically significant moderate effect sizes were apparent for the association between being bullied at ages three and five and obesity at age eleven.

**Table 4:** Bivariate associations between predictor variables (age 9 months to 7 years) and obesity in British children with and without intellectual disability at age 11

	Intellectual disability (OR (with 95 % CI)/p)	No intellectual disability (OR (with 95 % CI)/p)
Child Ethnicity		
White	1 (ref)	1 (ref)
Mixed	**2.74 (0.96–7.85)**	1.43** (1.46–1.77)
Indian	0.98 (0.15–6.70)	1.25 (0.94–1.68)
Pakistani/Bangladeshi	1.67 (0.98–2.84)	1.58*** (1.31–2.38)
Black/Black British	2.51*(1.17–5.36)	1.95*** (1.60–2.38)
Other	n/a	0.92 (0.647–1.32)
Child Birth Weight (high)	0.67 (0.9–1.16)	1.21*** (1.11–1.33)
Child Nutrition		
Early introduction of solids	1.35 (0.85–2.14)	1.38*** (1.24–1.52)
Low fruit consumption (age 5)	0.57* (0.33–0.98)	1.26*** (1.13–1.40)
Low fruit consumption (age 7)	1.29 (0.75–2.24)	1.24** (1.10–1.40)
High caloriebetween-meal snacks (age 5)	0.84 (0.53–1.33)	1.20** (1.06–1.36)
Does not eat breakfast every day (age 5)	1.13 (0.64–1.98)	1.82*** (1.51–2.20)
Does not eat breakfast every day (age 7)	1.80 (0.98–3.31)	1.52*** (1.26–1.82)
Child Participation in Sport		
Low participation (age 5)	0.83 (0.54–1.29)	1.39*** (1.28–1.52)
Low participation (age 7)	0.95 (0.57–1.60)	1.32*** (1.17–1.49)
TV Watching & Playing Computer Games		
High participation (age 3, TV only)	0.95 (0.60–1.51)	1.22** (1.09–1.37)
High participation (age 5, TV or computer games)	1.02 (0.65–1.61)	1.38*** (1.24–1.54)
High participation (age 7, TV or computer games)	1.32 (0.82–2.12)	1.30*** (1.16–1.45)
Exposure to Being Bullied		
Age 3	**1.91 (0.80–4.53)**	1.52* (1.07–2.15)
Age 5	**2.38 (0.95–5.96)**	1.31 (0.90–1.91)
Age 7	1.50 (0.49–4.57)	1.77*** (1.40–2.24)
Maternal Obesity		
No occasion	1 (ref)	1 (ref)
One or two occasions	1.53 (0.92–2.53)	2.74*** (2.44–3.07)
Three to five occasions	5.48*** (3.03–9.93)	3.76*** (3.33–4.25)
Maternal Smoking	0.88 (0.60–1.29)	1.32*** (1.21–1.43)
Maternal Educational Attainment		
NVQ (L3-5)	1 (ref)	1 (ref)
NVQ (L2)	1.52 (0.81–2.86)	1.55*** (1.40–1.72)
NVQ (L1 or none)	1.16 (0.65–2.10)	1.75*** (1.57–1.94)
Overseas qualifications only	3.95** (1.49–10.50)	1.41** (1.11–1.79)
Single Parent Family	1.26 (0.86–1.84)	1.28*** (1.18–1.40)
Household Income Poverty	1.47 (0.75–2.89)	1.41*** (1.28–1.54)
High Area Deprivation	1.23 (0.78–1.93)	1.53*** (1.30–1.81)

ORs in bold indicate medium or large effect size

*OR* odds ratio, *p* probability

* *p* < 0.05, ** *p* < 0.01, *** *p* < 0.001

Given the marked differences in sample sizes (and hence statistical power) between the subsamples of children with and without intellectual disability, between group comparisons should be made on estimates of the effect size (odds ratios) of associations rather than their statistical significance. While there was a stronger association between predictor variables and obesity among children without intellectual disability for 17 (61 %) of the 28 comparisons, this difference is not itself statistically significant (sign test *p* = 0.34, n.s.).

Table 5 presents information on the unique strength and statistical significance of the associations between predictor variables and child obesity at age 11 among children with intellectual disability for the subset of variables that had significant bivariate associations or moderate effect sizes with obesity at age 11 among this group of children. The two strongest predictors and the only predictors that were significantly associated with child obesity in this model were persistent maternal obesity and maternal education (only having overseas qualifications). However, moderate effect sizes were also apparent for child ethnicity (mixed and Black/Black British) and being bullied at age five.

**Table 5:** Multivariate analyses of association between predictor variables and risk of child obesity at age eleven among children with intellectual disability (*n* = 484)

Variable	OR (with 95 % CI)/p
Child Ethnicity	
White	1 (ref)
Mixed	**2.13 (0.69–6.64)**
Indian	1.02 (0.13–8.29)
Pakistani/Bangladeshi	1.81 (0.96–3.43)
Black/Black British	**2.33 (0.99–5.51)**
Maternal Obesity	
No occasion	1 (ref)
One or two occasions	1.50 (0.86–2.62)
Three to five occasions	7.18 (3.77–13.67)***
Exposure to Being Bullied	
Age 3	1.51 (0.37–6.20)
Age 5	**2.54 (0.74–8.77)**
Maternal Educational Attainment	
NVQ (L3-5)	1 (ref)
NVQ (L2)	1.61 (0.80–3.23)
NVQ (L1 or none)	1.15 (0.58–2.28)
Overseas qualifications only	4.31 (1.47–12.65)**
Low fruit consumption (age 5)	0.58 (0.32–1.05)

ORs in bold indicate medium or large effect size

*OR* odds ratio, *p* probability

* *p* < 0.05, ** *p* < 0.01, *** *p* < 0.001

Table 6 presents information on the unique strength and statistical significance of the association between the same predictor variables and child obesity at age 11 among children without intellectual disability. The largest effect sizes in these analyses were for persistent maternal obesity and child ethnicity (Black/Black British).

**Table 6:** Multivariate analyses of association between predictor variables and risk of child obesity at age eleven among children without intellectual disability (*n* = 13459)

Variable	OR (with 95 % CI)/p
Child ethnicity	
White	1 (ref)
Mixed	1.50 (1.21–1.87)***
Indian	1.38 (1.02–1.86)*
Pakistani/Bangladeshi	1.43 (1.18–1.74)***
Black/Black British	1.82 (1.48–2.24)***
Maternal Obesity	
No occasion	1 (ref)
One or two occasions	2.58 (2.30–2.90)***
Three to five occasions	3.71 (3.28–4.20)***
Exposure to Being Bullied	
Age 3	1.09 (0.77–1.55)
Age 5	1.05 (0.79–1.41)
Maternal educational attainment	
NVQ (L3-5)	1 (ref)
NVQ (L2)	1.47 (1.33–1.64)***
NVQ (L1 or none)	1.58 (1.41–1.77)***
Overseas qualifications only	1.18 (0.91–1.53)
Low fruit consumption (age 5)	1.15* (1.02–1.28)

ORs in bold indicate medium or large effect size

*OR* odds ratio, *p* probability

* *p* < 0.05, ** *p* < 0.01, *** *p* < 0.001

Given the unexpected significance of maternal education we undertook post hoc analyses to identify the ethnic characteristics of this group and investigate whether this association was also apparent at earlier ages. The small group (*n* = 25) of mothers of children with intellectual disability who only had overseas qualifications described themselves most commonly as being of Black/Black British (36 %), Pakistani/Bangladeshi (32 %) and White (29 %) heritage. Higher rates of obesity in children were also apparent at age 5 (15 v 12 %, OR = 1.34 [0.99–1.83], Fisher’s exact p <0.05) and seven (15 v 13 %, OR = 1.23 [0.90–1.23], Fisher’s exact p n.s.), although the effect sizes were small and at age seven non-significant.

## Discussion

Our results indicated that children with intellectual disabilities were significantly more likely than children without intellectual disabilities to be obese at ages five, seven and eleven. At ages five and seven increased risk of obesity among children with intellectual disabilities was only apparent among boys. At age eleven increased risk of obesity among children with intellectual disabilities was also apparent among girls. Among children with intellectual disability risk of obesity at ages five and seven was associated with male gender. At age eleven risk of child obesity was associated with persistent maternal obesity, maternal education (only having overseas qualifications), child ethnicity (mixed and Black/Black British) and being bullied at age five. The first two factors showed strong effect sizes and were statistically significant, the latter two, while not statistically significant, showed moderate effect sizes.

The results of the present study add to the existing literature on obesity among children with intellectual disabilities in three ways. First, it provides estimates of the risk of obesity associated with intellectual disability in a contemporary population-based sample of children with and without intellectual disability. The reported effect sizes are consistent with those reported in previous population-based samples in the UK [23], Australia [16] and the US [27].

Second, the data identify marked age effects, with obesity in children with intellectual disability rising from 15 to 17 % at ages five and seven to 31 % at age 11. That nearly one in three 11 year old children with intellectual disability in the UK are obese is of considerable concern. Similar age-related trends in the increase of obesity were also evident among children without intellectual disability (12–13 % at ages five and seven to 21 % at age 11). There were apparent age-related trends in the association between gender and obesity, with increased rates of obesity among boys at younger ages, but no difference at age 11. These effects may account for the noted inconsistency in the existing literature on the association between gender and risk of obesity among children with intellectual disabilities [15]. As noted in the introduction, higher rates of obesity among adults with intellectual disability have consistently been reported among women [32–34], a finding also reported in several studies of children/young people with intellectual disability [16–18, 20–22, 26]. Further research is required to identify the extent to which age moderates the association between gender and obesity among people with intellectual disabilities.

Finally, this is, to our knowledge, the first study to identify an association between child ethnicity, maternal migrant status and exposure to bullying and obesity among children with intellectual disability. The latter is consistent with research in the general population on the association between exposure to violence in childhood and subsequent obesity [62] and the findings of the small number of studies that have investigated the association between exposure to bullying in childhood and later obesity [63–65].

However, as in all studies, there are limitations that need to be taken into account when considering these findings. First, while having access to a large, longitudinal dataset is an asset, datasets (such as the MCS) that are designed for multiple purposes commonly utilise abbreviated forms of measures such as the abbreviated scales of cognitive functioning (rather than complete IQ tests) used in the MCS. Second, while the overall sample was relatively large, it was of insufficient size to examine the extent to which our results generalized to children with severe intellectual disability. It is important, therefore, to keep in mind, therefore, that our results regarding intellectual disability primarily relate children with mild or moderate intellectual disability.

Nevertheless, our results are consistent with previous population-based studies in indicating that children with intellectual disability, at least in high-income countries, are a high-risk group for the development of obesity accounting for 5–6 % of all obese children. Interventions to reduce the prevalence and inequities in the distribution of child obesity will need to take account of the specific situation of this ‘high risk’ group of children.

## Abbreviations

ADHD, attention deficit hyperactivity disorder; BAS, British Ability Scales; BMI, body mass index; GCSE, General Certificate of Secondary Education; IQ, intelligence quotient; MCS, Millennium Cohort Study; NFER, National Foundation for Educational Research; NVQ, National Vocational Qualifications; OECD, Organisation for Economic Co-operation and Development; OR, odds ratio; TV, television; UCL, University College London; UK, United Kingdom
